# Combined Cutaneous Therapy Using Biocompatible Metal-Organic Frameworks

**DOI:** 10.3390/nano10122296

**Published:** 2020-11-25

**Authors:** Seyed Dariush Taherzade, Sara Rojas, Janet Soleimannejad, Patricia Horcajada

**Affiliations:** 1 Advanced Porous Materials Unit (APMU), IMDEA Energy, Av. Ramón de la Sagra 3, 28935 Móstoles-Madrid, Spain; d.taherzade@ut.ac.ir (S.D.T.); sara.rojas@imdea.org (S.R.); 2 School of Chemistry, College of Science, University of Tehran, P.O. Box 14155-6455, Tehran 1417614411, Iran; janet_soleimannejad@khayam.ut.ac.ir

**Keywords:** metal-organic frameworks, combined therapy, azelaic acid, nicotinamide, cutaneous treatment

## Abstract

Combined therapies emerge as an interesting tool to overcome limitations of traditional pharmacological treatments (efficiency, side effects). Among other materials, metal-organic frameworks (MOFs) offer versatilities for the accommodation of multiple and complementary active pharmaceutical ingredients (APIs): accessible large porosity, availability of functionalization sites, and biocompatibility. Here, we propose topical patches based on water-stable and biosafe Fe carboxylate MOFs (MIL-100 and MIL-127), the biopolymer polyvinyl alcohol (PVA) and two co-encapsulated drugs used in skin disorders (azelaic acid (AzA) as antibiotic, and nicotinamide (Nic) as anti-inflammatory), in order to develop an advanced cutaneous combined therapy. Exceptional MOF drug contents were reached (total amount 77.4 and 48.1 wt.% for MIL-100 and MIL-127, respectively), while an almost complete release of both drugs was achieved after 24 h, adapted to cutaneous delivery. The prepared cutaneous PVA-MOF formulations are safe and maintain the high drug-loading capacity (total drug content of 38.8 and 24.2 wt.% for MIL-100 and MIL-127, respectively), while allowing a controlled delivery of their cargoes, permeating through the skin to the active target sites. The total amount of drug retained or diffused through the skin is within the range (Nic), or even better (AzA) than commercial formulations. The presented results make these drug combined formulations promising candidates for new cutaneous devices for skin treatment.

## 1. Introduction

A wide range of active pharmaceutical ingredients (APIs) targets the skin for therapeutics or cosmetic purposes [1,2,3]. Since ancient times, topical delivery has been considered as a route for the delivery of pharmaceuticals. Skin diseases can be treated both by topical and oral medications. As an advantage, the topical ones avoid the hepatic first-pass metabolism and often prevent the side effects of systemic drug absorption [4]. One of the most common skin disorders, acne vulgaris, is a long-term skin disease that can be temporary or permanent, and even lead to the formation of painful cysts and nodules in severe cases [5]. In 2015, acne affected 633 million people globally, being among the top 10 most prevalent diseases worldwide [6,7]. The primary cause of acne is an obstruction of the pilosebaceous canal because of at least four pathogenic factors (proliferation of follicular epithelium, sebum production, infection, and inflammation) [8]. In this sense, there are many different anti-acne therapies (e.g., anti-androgen, oral antibiotics, antiseborrheics, hormonal treatments, benzoyl peroxide) [9]. Among them, a number of small and simple APIs, either alone or in combination, have recently emerged as promising treatments of common skin disorders, such as azelaic acid (AzA, or nonanedioic acid) and nicotinamide (Nic, also known as niacinamide) [10,11,12]. AzA is a topical antimicrobial and anti-inflammatory agent, which further reduces keratin production, normally used to treat acne and also rosacea [13,14], being well-tolerated at various concentrations (from 1 to 20 wt.%). However, the limited AzA skin penetration across the *stratum corneum* of its commercialized products (~4% when using Skinoren^®^–20%, corresponding to ~130 µg) [15] and their commonly recognized side effects (skin irritation) [16] make them therapeutically ineffective and dangerous. Further, additional issues such as the bacterial resistance and the poor concentration of this topical antibiotic have been lately raised. In this sense, it has been suggested that repeated applications of AzA preparations could maintain the follicular concentration of AzA at biologically active levels [17]. On the other hand, Nic is a newly approved anti-acne drug with a potent anti-inflammatory effect, that is extremely well-tolerated by facial skin [18]. However, Nic suffers from poor skin retention and permeation (~1% after 72 h) [19] because of its high aqueous solubility and low partition coefficient [20]. Several strategies to increase Nic flux to the skin (i.e., improving drug solubility through the interaction with different skin permeation enhancers, or increasing the drug saturation within the formulation) have been reported [21]. Nevertheless, it has been suggested that the use of high doses of Nic can lead to lesions on the skin, toxic amblyopia, and hypotensive reactions [22]. Thus, new dosage forms and matrix design of AzA and Nic are currently required to improve their skin retention (within the epidermis and dermis, more particularly in the pilosebaceous unit) [23] and penetration in the targeted areas, and lately their therapeutic efficacy.

The combination of two agents can overcome toxicity and other side effects associated with high dose of single drugs, by either countering biological compensation, sparing doses on each compound, or accessing to contest-specific multi-target mechanisms [24,25]. In this regards, the combination of different APIs in the same formulation has proven a superior anti-acne effect than individuals (AzA 20% + glycolic acid [26], AzA 5% + clindamycin 2% [27], erythromycin 2% [28], or AzA + Nic 10%) [29]. Despite its interest, providing a constant and uniform concentration of drugs in the target site still remains a challenge [30]. In this sense, metal-organic frameworks (MOFs) have recently emerged as great candidates for drug delivery applications, not only providing progressive release of the APIs but also modifying their biodistribution [31,32,33]. MOFs are comprised of inorganic nodes and organic polycomplexant linkers that assemble into multidimensional periodic lattices through coordination bonds [34]. Although there are not many works in the literature concerning MOFs as platform for the simultaneous co-encapsulation of APIs (i.e., drugs, metals, gases, biomolecules), in recent years, some research groups have proposed these systems as advanced drug delivery therapies, theranosis, or multiple detection bioimaging systems [35,36]. Among them, only two studies report the use of MOFs for the simultaneous co-delivery of APIs in cutaneous applications. The first, proposes two nanometric MOFs based on Ni^2+^ and Cu^2+^ combined with poly(tetrafluoroethylene) forming patches for the local co-delivery of the bioactive gas NO and the antibiotic drug metronidazole [37]. In the second study, some of us reported a biocompatible MOF, based on an antibiotic metal (Zn^2+^) and AzA, with a high chemical stability associated to a progressive release of their active components [38].

Following this trend in improving cutaneous treatments with the co-release of APIs, the co-encapsulation and simultaneous release of two organic drugs (AzA and Nic) in cutaneous conditions will be here investigated for the first time using two selected biocompatible and stable Fe(III) carboxylate MOFs (MIL-100 and MIL-127; see below) [39,40], preparing a specific cutaneous polymer-based formulation and evaluating its skin permeation with the aim to develop an advanced cutaneous combined therapy.

## 2. Materials and Methods

### 2.1. Materials and Reagents

All reactants were commercially obtained and used without further purification. Ethyl ester 1,3,5-benzenetricarboxylic (97%) was obtained from Alfa Aesar™. Azelaic acid (AzA, 98%), nicotinamide (Nic, 99%), polyvinyl alcohol (PVA, 98%, MW: 180,000 g·mol^−1^), and 5-nitroisophthalic acid (98%) were purchased from Sigma Aldrich. The adhesive film (Leukoflex^®^) was purchased from a local drug store. The synthesis of the starting materials was performed following similar procedures previously reported:

#### 2.1.1. Synthesis of 3,3′,5,5′-Azobenzenetetracarboxylic Acid (H_4_TazBz)

5-Nitroisophthalic acid (19 g, 90 mmol) and NaOH (50 g, 1250 mmol) were mixed in 250 mL of distilled water (dH_2_O), placed into a 1-L 3-neck round bottom flask and stirred vigorously at 60 °C. 100 g of D-glucose was dissolved in 150 mL of dH_2_O and added to this slurry solution. After cooling down to room temperature (RT), airflow was bubbled into the brown mixture for 4 h under stirring. After cooling down in an ice-bath, the disodium salt was recovered by filtration and washed with a small amount of cold dH_2_O. The resulting yellow solid was then dissolved in 200 mL of dH_2_O and this solution was acidified to pH = 1 by the addition of HCl (37%). The resulting orange solid was recovered by filtration, washed with dH_2_O, and dried at 100 °C under vacuum [40].

#### 2.1.2. Synthesis of [Fe_3_O(H_2_O)_2_OH(C_9_H_3_O_6_)_2_]·nH_2_O (MIL-100)

Ethyl ester 1,3,5-benzenetricarboxylic (1.94 g, 6.6 mmol) and FeCl_3_·6H_2_O (2.70 g, 10 mmol) were poured into a Teflon-lined autoclave (125 mL) with 50 mL of dH_2_O. The autoclave was heated at 130 °C for 72 h. After cooling to RT, the obtained solid was recovered by filtration. A total of 1 g of the obtained solid was refluxed first in bi-distilled water (Milli-Q water, 150 mL, 3 h) and then in absolute ethanol (150 mL, 3 h). The solid was then suspended in a KF solution (0.1 M, 50 mL, 3 h, 70 °C) and hot filtered. Finally, it was suspended in 150 mL of Milli-Q water (2 h, RT) and recovered by filtration [41].

#### 2.1.3. Synthesis of [Fe_3_O(OH)_0.88_Cl_0.12_(C_16_N_2_O_8_H_6_)_1.5_(H_2_O)_3_]·n(H_2_O) (MIL-127)

NaOH (0.316 g, 7.90 mmol) was dissolved in 0.6 mL of dH_2_O. Separately, H_4_TazBz (1.15 g, 3.21 mmol) was dissolved in 6.3 mL of 2-propanol. Then, both solutions were mixed. FeCl_3_·6H_2_O (1.758 g, 6.50 mmol) was dissolved in 5.0 mL of 2-propanol and then, added to the first batch. The final mixture was refluxed for 24 h. The final obtained brown solid was recovered by filtration and washed with 320 mL of dH_2_O and 320 mL of ethanol [40].

### 2.2. Experimental Techniques

Thermogravimetric analyses (TGA) were performed using a Perkin Elmer STA 6000 with an air flow of 100 mL·min^−1^ and a ramp of 5 °C·min^−1^. Fourier transformed infrared spectroscopy (FT-IR) analyses were carried out on a Thermo Nicolet 6700 spectrometer (Thermo, Waltham, MA, USA). An Autosorb iQ2 (Quantachrome Instruments, Boynton Beach, FL, USA) was used for N_2_ sorption analysis. Before any measurement, solids were outgassed (10^−2^ mbar) using different conditions: 130 °C/3 h for MIL-100 and NicAzA@MIL-100, and 160 °C/16 h for MIL-127 and NicAzA@MIL-127. Elemental analysis (EA) was determined using a FLASH 2000 (Thermoscientific, Waltham, MA, USA). Routine X-ray powder diffraction (XRPD) patterns were collected using a conventional PANalytical Empyrean powder diffractometer (PANalytical, Lelyweg, The Netherlands, *θ*–2*θ*) using λCu K_α1_, and K_α2_ radiation (λ = 1.54051 and 1.54433 Å). The XRD diagrams were carried out with a 2*θ* scan between 3–30° with a step size of 0.013° and a scanning speed of 0.1°·s^−1^. The particle size determinations were performed using a Malvern Nano-ZS, Zetasizer Nano series. Around 1 mg of sample was dispersed in 10 mL in dH_2_O using an ultrasound tip (Digital Sonifer 450, Branson, with a 20% of amplitude for 1 min). Each formulation was analyzed in triplicate (n = 3). The morphology of MOFs particles was characterized by Scanning Electron Microscopy (SEM) using aTM1000 from Hitachi.

### 2.3. High Performance Liquid Chromatography (HPLC) Measurement Conditions

Quantification of trimesic acid (H_3_BTC), H_4_TazBz, AzA and Nic was performed using HPLC using a reversed phase Jasco LC-4000 series system, equipped with a PDA detector MD-4015 and a multisampler AS-4150 controlled by ChromNav software (Jasco Inc., Easton, MD, USA). A Purple ODS reverse-phase column (5 µm, 4.6 × 150 mm^2^ Análisis Vínicos) was employed. Note here that AzA was analyzed after its derivatization (der-AzA), as previously described [42]. The mobile phase consisted of a 50:50 solution (*v*/*v*) of buffer (0.04 M, pH = 2.5) and methanol (MeOH) for H_3_BTC and H_4_TazBz; 20:80 solution (*v*/*v*) acetonitrile:buffer (0.5 mM, pH = 4) for der-AzA; and 5:95 solution (*v*/*v*) acetonitrile:buffer pH = 4 for Nic. The injection volume was set at 30 µL with a flow rate of 1 mL min^−1^ and the column temperature fixed at 25 °C (H_3_BTC and H_4_TazBz) and 40 °C (der-AzA and Nic). The standard calibration curves showed a good correlation coefficient ≥0.99. The chromatogram of standard solutions showed a retention time (rt) of 3.51 min (identified as H_3_BTC, λmax at 225 nm, Figure S1), 21.36 min (identified as H_4_TazBz, λmax at 205 nm, Figure S2), 9.12 min (identified as der-AzA, λmax at 255 nm, Figure S3), and 5.56 min (identified as Nic, λmax at 213 nm, Figure S4).

#### Buffers Preparation

(a) Buffer 0.04 M, pH = 2.5: NaH_2_PO_4_ (2.4 g, 0.02 mol) and Na_2_HPO_4_ (2.84 g, 0.02 mol) were dissolved in 1 L of Milli-Q water. The pH was then adjusted to 2.5 with H_3_PO_4_ (≥85%).

(b) Buffer 0.5 mM, pH = 4: NH_4_HCO_3_ (39.53 mg, 0.5 mmol) was dissolved in 1 L of Milli-Q water. The pH was then adjusted to 4 with HCl 1 M.

### 2.4. Drugs Encapsulation Studies

#### 2.4.1. Combined Encapsulation of Drugs (AzA and Nic)

Prior to the AzA entrapping, the azelate salt was prepared in order to improve its aqueous solubility and, thus, its encapsulation rate. This step is crucial to improve the AzA encapsulation since its solubility in dH_2_O is limited (2.14 g·L^−1^ at 25 °C) [43]. Further, it has been previously demonstrated that its solubility (and not its hydrophilic nature) is the rate-limiting factor in skin absorption and permeation [44]. A deprotonated form [Az^2−^] of the acid (pKa1 = 4.55 and pKa2 = 5.60) was prepared by dissolving AzA (0.10 g, 0.53 mmol) in 50 mL of a NaOH aqueous solution (0.02 M) using a molar ratio 1:2 [45,46]. The final pH was adjusted to 6 with HCl (0.01 M). After the disodium azelate solution was prepared, Nic (0.50 g, 4.09 mmol) was added to obtain the AzA and Nic solution.

In the following, 60 mg of Fe-MOF (0.09 mmol of MIL-100, or 0.07 mmol of MIL-127) were suspended in 20 mL of the previously prepared AzA and Nic solution with a weight ratio of 1:5:0.6 (AzA: Nic: MOF) under stirring for 24 h at RT. The drug-loaded materials (NicAzA@MIL-100 and NicAzA@MIL-127) were recovered by filtration and washed with dH_2_O (3 × 5 mL). Finally, it should be pointed that altering the initial concentration of the drugs or using several consecutive impregnations did not improve the drugs-loading capacity. Note here that the amount of encapsulated Nic and AzA was performed by EA, TGA, and HPLC, and the combination of these techniques confirmed the encapsulation of the AzA disodium salt. All the experiments were carried out in triplicate (n = 3).

#### 2.4.2. Encapsulation of a Single Drug (AzA or Nic)

The same previously described procedure was followed in order to incorporate a single drug in the Fe-MOFs. AzA (0.10 g, 0.53 mmol) was solved in 50 mL of a water solution of NaOH (0.02 M). The final pH was adjusted to 6 with HCl (0.01 M), and 60 mg of Fe-MOF (0.09 mmol of MIL-100, or 0.07 mmol of MIL-127) were suspended in this AzA solution with a weight ratio of 1:0.6 (AzA:MOF) under stirring for 24 h at RT. The AzA-loaded materials (AzA@MIL-100 and AzA@MIL-127) were recovered by filtration, and cleaned with dH_2_O (3 × 5 mL). Nic (0.5 g, 4.09 mmol) was solved in 2 mL of dH_2_O (pH adjusted to 6 with NaOH, 0.01 M) and 60 mg of Fe-MOF (0.09 mmol of MIL-100, or 0.07 mmol of MIL-127) were suspended in this Nic solution with a weight ratio of 5:0.6 (Nic:MOF) under stirring for 24 h at RT. The Nic-loaded materials (Nic@MIL-100 and Nic@MIL-127) were recovered by filtration, and washed with dH_2_O (3 × 5 mL). All the experiments were carried out in triplicate (n = 3).

The amounts of adsorbed drugs were quantified by HPLC, TGA, and EA (see Table S1).

The drugs-loading content was also described by the encapsulation efficiency (*EE*), where $EE\left(\%\right)=\frac{Dl}{Dt}\cdot 100$; *Dt* is the initial amount of drug present in the starting impregnation solution and *Dl* is the amount of loaded drug [47].

Additionally, the amount of the possible ligand leached during the drugs encapsulation processes was measured by HPLC, determining the MOF stability. Pristine Fe-MOF and drug/s loaded Fe-MOFs were fully characterized by XRPD, FTIR, and N_2_ sorption measurements (Figures S5–S10).

### 2.5. Drug Delivery Studies

Drugs-containing MOFs (50 mg, NicAzA@MIL-100 or NiAzA@MIL-127) were placed in 40 mL of dH_2_O under stirring during 24 h at 32 °C, simulating dermal conditions. At different incubation times (5, 10, 15, 30, 45 min, 1, 2, 3, 10 and 24 h), 20 mL of supernatant was recovered by centrifugation (15.000 rpm, 10 min) and replaced with the same volume of fresh dH_2_O at 32 °C (considering the poor solubility of ligands, always working under sink conditions). This procedure was performed in triplicate for each compound. The amounts of released drugs and leached ligands were determined by HPLC. Note here that the amount of released AzA was quantified considering all azelate species.

### 2.6. Nic and AzA Skin Permeation Test

#### 2.6.1. Patches Preparation

Composite patches were prepared by an easy and rapid compression molding approach. Total of 50 mg of Nic and AzA-loaded Fe-MOFs and 50 mg of polyvinyl alcohol (PVA) were mixed and manually milled together. The resulting powder was readily put onto a 12-mm diameter-size wafer mold and pressed at 1 ton for 1 min, following a similar previously described procedure [48]. The corresponding control patches (NicAzA@A and NicAzA@B) were prepared from a mixture of the corresponding AzA and Nic found in the NicAzA@MIL-100 and NicAz@MIL-127 formulations, named NicAzA@A and NiAzA@B, respectively (Table S5). The as-prepared patches were characterized by XRPD (Figure S13).

#### 2.6.2. Skin Irritation Test

A 12-mm diameter pellet, prepared as previously described, was applied to the skin of the anterior cutaneous of forearm of 6 human volunteers for up to 8 h. Treatment site is assessed by means of the presence of redness (irritation) at 4 and 8 h after patch removal using a 4-point scale (0, no reaction; + weakly positive reaction; ++, moderate positive reaction, +++ strongly positive reaction). After the treatment, the volunteers did not feel itching sensation, warm, or humidity. No ulcers or skin injuries appeared. The skin had always a healthy tissue appearance.

#### 2.6.3. Nic and AzA Ex Vivo Permeation Tests

Fresh porcine ears were obtained from a local slaughterhouse and after cleaning, the outer region of the ear was cut. Afterwards, the skin was dermatomed to 1.2 mm (using a Brown air Dermatome Zimmer) and stored at −20 °C. Skin samples were placed in a simple model of Franz diffusion chambers with the inner area of the stratum corneous faced to the donor compartment and the dermis to the receptor one, leaving an available diffusion surface of 0.625 cm^2^. The receptor compartment was filled with 5.5 mL of PBS (pH = 7.4, Sigma Aldrich, St. Louis, MO, USA), and kept between 32 °C for 24 h. Both different formulation patches together with their respective controls (NicAz@MIL-100_patches, NicAc@MIL-127_patches, NicAz@A and NicAz@B) were readily placed on the top of the *stratum corneum* (final patches contact surface = 0.41 cm^2^) and covered by an impermeable polymeric film (Leukoflex^®^). Ex vivo permeation studies were performed for 24 h, collecting 1 mL of receptor fluid after 1, 4, 8, and 24 h, being replaced immediately with 1 mL of fresh medium at the same temperature. The obtained aliquots were analyzed by HPLC in order to quantify the Nic, AzA, H_3_BTC, and H_4_TazBz content, as previously described. The diffusion flux (J) was calculated using the following equations:

*J* = *m*/*At*, (1)

where *J* is the flux of a mass of compound *m* moving through a cross-sectional area *A* during *t*. It has to be noted that identical Nic and AzA content were used in the MOF-based patch and the corresponding control patch (Table S5). All of the experiments were carried out in quadruple for each experimental data. Finally, the patches remaining on the skin surface were removed, cleaning the skin with PBS (pH = 7.4). Subsequently, the skin was dried and cut for AzA and Nic quantification following a similar extraction procedure previously described [49]. The normality of data distribution was tested by one-way ANOVA test. Data are shown as the mean and the standard deviation. A value of *p* < 0.05 was considered statistically significant.

#### 2.6.4. Nic and AzA Extraction from the Skin

A mixture of 0.5 mL of sodium dodecyl sulphate (SDS, 0.01 M) and 1 mL of PBS (0.5 M, pH = 7) was added to ca. 100–120 mg of skin in an assay tube. This mixture was homogenized by grinding with a Potter–Elvehjem tissue grinder, and then sonicated for 1 h. About 10 mL of methanol was added and the mixture was stirred for 2 h. Then, samples were centrifuged at 10.000 rpm for 10 min and filtered through 0.2-µm sterile syringe filter. Finally, the supernatants were evaporated and the dry residue was dissolved in 4 mL of methanol and 4 mL of acetonitrile. Prior to the injection into the HPLC system, samples were diluted in the mobile phase. All the extraction studies were carried out in four-fold (n = 4).

#### 2.6.5. Buffer Preparation

(c) Buffer 0.5 M, pH = 7: NaH_2_PO_4_ (30 g, 0.25 mol), and Na_2_HPO_4_ (35.5 g, 0.25 mol) were dissolved in 1 L of Milli-Q water. The pH was then adjusted to 7 with H_3_PO_4_ (≥85%).

## 3. Results and Discussion

### 3.1. Drug Encapsulation

**Figure 1:**
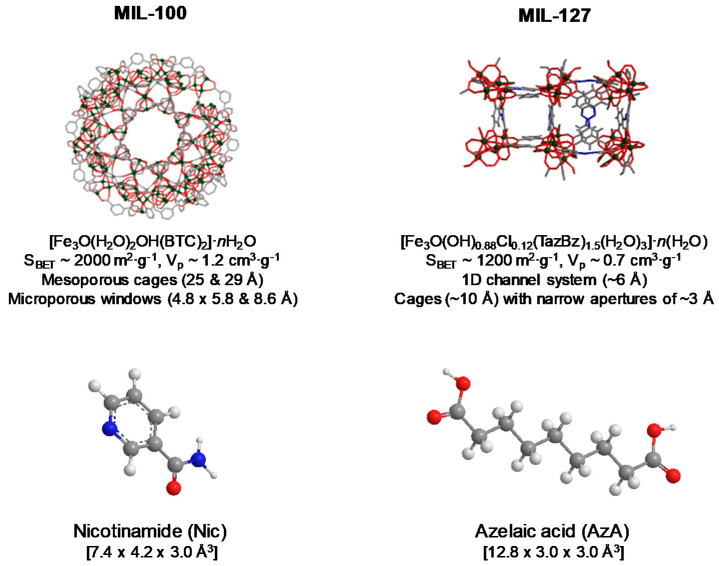
Schematic view of the structure of MIL-100 and MIL-127 (iron polyhedra, nitrogen, oxygen, and carbon are represented in green, blue, red, and gray, respectively; hydrogen atoms are omitted for clarity), their formulas, and the description of their structure and porosity. Nic and AzA molecules and their dimensions (size calculated from Vesta free software considering van der Waals radii) are also given.

For this study, we have selected the more hydrophilic mesoporous MIL-100(Fe), [Fe_3_O(H_2_O)_2_OH(BTC)_2_]·*n*H_2_O (BTC: 1,3,5-benzenetricarboxylate or trimesate) based on iron (III) octahedra trimers and trimesate anions leading to a very high porosity (Brunauer, Emmett, and Teller surface area-S_BET_~2000 m^2^·g^−1^, V_p_~1.2 cm^3^·g^−1^, particle size from ~30 to several hundred nanometres), associated with two types of mesoporous cages (25 and 29 Å) accessible through microporous windows (*ca*. 4.8 × 5.8 and 8.6 Å, respectively) [39]; and the microporous hydrophilic/hydrophobic MIL-127(Fe) structure, [Fe_3_O(OH)_0.88_Cl_0.12_(TazBz)_1.5_(H_2_O)_3_]·*n*(H_2_O) (TazBz: 3,3′,5,5′-azobenzenetetracarboxylate, S_BET_~1200 m^2^·g^−1^, V_p_~0.7 cm^3^·g^−1^, particle size 0.9 ± 0.3 µm) based on iron(III) octahedra trimers and TazBz^4−^ anions, associated with two types of pores, namely, an accessible hydrophobic one-dimensional (1D) channel system (~6 Å) and cages (~10 Å), accessible to narrow apertures of ~3 Å (Figure 1) [40]. Aside from their high porosity, MIL-100 and MIL-127 are based on non-toxic components (Fe(III), BTC and TazBz), having proven no sign of toxicity against different cell lines (e.g., HeLa, J774, A549, Calu-3, HepG2, Hep3B) [50,51] and even upon the administration of very high doses to rats (i.e., intravenous administration of 220 mg·Kg^−1^ of MIL-100 [52]; or oral administration of 1 g·kg^−1^ of MIL-127) [53]. Further, these two Fe-MOFs have shown to be safe and stable platforms for the release of various drugs with different chemical nature (e.g., NO [54], tetracycline [55], aspirin [56], ibuprofen [57], docetaxel [58], doxorubicin [32], RAPTA-C [59], and caffeine [60], among others).

Drugs were successfully encapsulated in both Fe-based MOFs following a simple and completely bio-friendly impregnation method, based on the soaking of the porous materials in an aqueous solution of a drugs mixture (AzA and Nic). It should be noted here that an initial AzA:Nic ratio of 1:5 was selected considering that commercial formulation used an AzA:Nic ratio from 1:4 (topical; Sincerus Florida LCC) to 1:100 (oral; VP-ZEL) [61]. Further, these forms are administered following a different route, being therefore associated to a different bioavailability (~3 vs. 60% of cutaneous and orally administered AzA are excreted with the urine only after 12 h of the treatment, respectively) [62].

**Figure 2:**
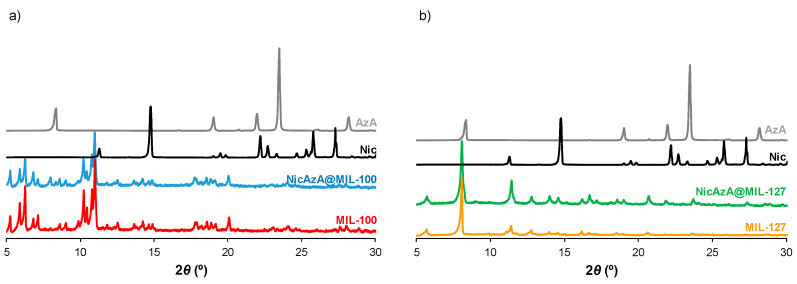
XRPD patterns of (**a**) MIL-100, NicAzA@MIL-100; and (**b**) MIL-127, NicAzA@MIL-127, as well as free Nic and AzA.

**Figure 3:**
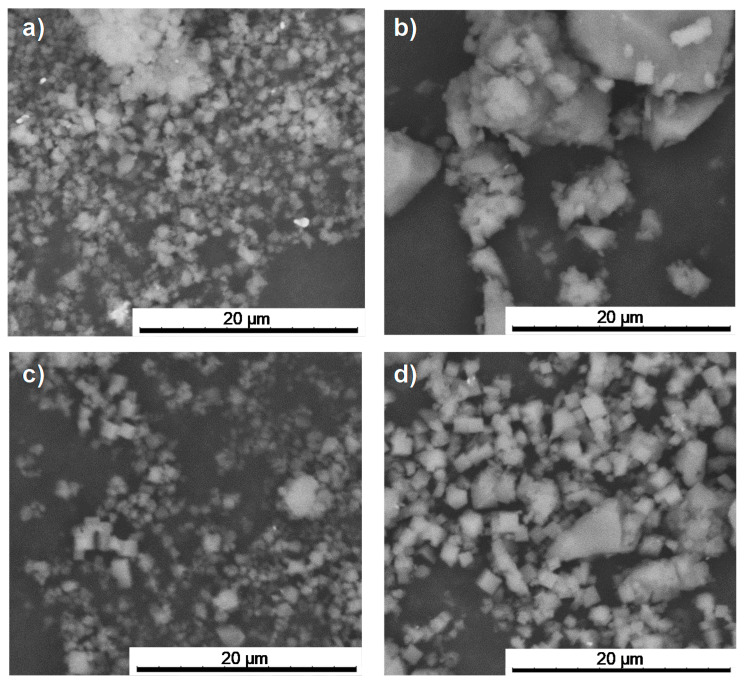
SEM images of the prsitine (**a**) MIL-100 and (**c**) MIL-127; and the corresponding drugs-loaded materials (**b**) NicAzA@MIL-100 and (**d**) NicAzA@MIL-127.

After drug encapsulation, X-ray powder diffraction (XRPD) patterns evidence that the drug-loading process does not alter the crystalline structure of the porous materials (Figure 2). In addition, the absence of Bragg peaks corresponding to free Nic and AzA rules out the presence of free recrystallized drug out of the pores. Upon encapsulation of the drugs, particle size and morphology were maintained (Figure 3, Table S2). The drugs content was estimated by combining thermogravimetric analysis (TGA), high-performance liquid chromatography (HPLC), and elemental analysis (EA, Table S1). Remarkably, high drugs loadings were obtained for both MOFs (Table 1). When comparing both porous materials, higher drugs loadings were obtained for MIL-100, in agreement with its higher accessible porosity (Figure S7, Table S3). When comparing the drugs content obtained here (58.4 wt.% Nic and 19.1 wt.% AzA for MIL-100; and 34.1 wt.% Nic and 14.2 wt.% AzA for MIL-127) with other commercial formulations (10–20 wt.% of AzA and/or 10 wt.% of Nic) [26,29], a higher encapsulation efficiency in both porous materials is achieved in the current study, with a total impressive drug content of 77.4 wt% and 48.1 wt.% for MIL-100 and MIL-127, respectively. These values are among the highest reported so far in MIL-100 (e.g., 50 wt.% of caffeine [60], 25 wt.% of aspirin [56], and 31 wt.% of ibuprofen) [57]. By also comparing the drug by drug loadings, similar contents are obtained for AzA (19.1 wt.% for MIL-100 and 14.2 wt.% for MIL-127), observing major differences in Nic cargoes (58.4 wt.% for MIL-100 and 34.1 wt.% for MIL-127). Further, it should be highlighted that there are no reports dealing with the encapsulation of Nic in MOFs and only one work related with the incorporation of AzA in the BioMIL-5 ([Zn(C_9_O_4_H_14_)]) as constitutive ligand (not adsorbed) [38]. For comparison, studies encapsulating one single drug (AzA or Nic) were performed for both MOFs (Table 1), leading to the successful entrapment of AzA or Nic in MIL-100 (9.1 or 15.8 wt.%, respectively) and MIL-127 (34.3 or 16.0 wt.%, respectively). Except when using AzA+MIL-127 or Nic+MIL-127 combinations (34.3 and 16.0 wt.%, respectively), lower encapsulation rates were reached. These results proved that AzA and Nic presence mutually enhances their adsorption capability, as previously described in other adsorption processes (i.e., adsorption of dyes in PCN-222 MOF) [63]. We believe that the large porosity of MIL-100 and MIL-127 facilitates the fast adsorption and makes the co-adsorption possible. Further, both structural characteristic and hydrophobic/hydrophilic balance between drugs and MOFs, are key parameters on the adsorption of drugs. As the vast majority of MOFs, MIL-100, and MIL-127 can be considered amphiphilic solids, including a hydrophilic inorganic part and a more hydrophobic organic fraction. However, we could comparatively propose that MIL-100 structure is the more hydrophilic, exhibiting polar hydroxyl group in their accessible windows (~4.8 × 5.8 Å^2^ and 8.6 Å) and more metallic clusters per ligand, while MIL-127 presents accessible hydrophobic 1D channels (~6 Å) and more hydrophilic cages (~10 Å), only accessible from narrow apertures of ~3 Å. Thus, if we consider the octanol/water partition coefficient of AzA and Nic (log Kow = 1.57 and −0.38, respectively), we can assume that Nic presents a more hydrophilic character than AzA (even considering its sodium salt). With the exception of AzA@MIL-127, drugs co-encapsulation leads to higher loadings than when adsorbing a single drug. This could be explained by a modification of the hydrophilic/hydrophobic character of the MOFs as a consequence of the presence of the drug. The higher loading of single AzA in the more lipophilic MIL-127 might be a consequence of the more hydrophobic character of this drug (single 34.3 wt.% vs. co-loading 14.2 wt.%).

The incorporation of Nic and AzA into the MIL-100 and MIL-127 cavities was further demonstrated by the dramatic reduction of the N_2_ sorption capacity of the MOFs and their pore size (even without considering the drugs loading, Figure S7, Table S3). In contrast with NicAzA@MIL-100, exhibiting an absence of porosity accessible to nitrogen upon drugs insertion, in the MIL-127 material there is still some residual porosity after the drugs encapsulation process (S_BET_ = 710 m^2^·g^−1^, V_p_ = 0.33 cm^3^·g^−1^). This suggests that the maximum drug loading is not reached in MIL-127 or that some cages are not accessible to the drugs but only to nitrogen. This could be related to a selective adsorption of both drugs that occurs only within the hydrophobic channels of MIL-127 (~6 Å) which correspond to ca. 42% of the total porosity. In contrast, the dimensions of both AzA (12.8 × 3.0 × 3.0 Å^3^) and Nic (7.4 × 4.2 × 3.0 Å^3^, Figure 1) might prevent their insertion into the hydrophilic cages of MIL-127, accessible only through small apertures of ~3 Å.

Finally, considering that the main difference between both drugs-loaded MOFs is related with the amount of adsorbed Nic (58.4 wt.% in MIL-100 and 34.1 wt.% in MIL-127), the influence of the hydrophobic/hydrophilic balance between MOFs (MIL-100 more hydrophilic than MIL-127) and drugs (hydrophilic Nic and hydrophobic AzA) and the more open structure in MIL-100 framework might be at the origin of these dissimilarities. Further, apart from important geometrical parameters and the hydrophobic/hydrophilic balance, the mutual enhancement of individual adsorption by MOFs is of major importance (see below).

### 3.2. Drug Release

**Figure 4:**
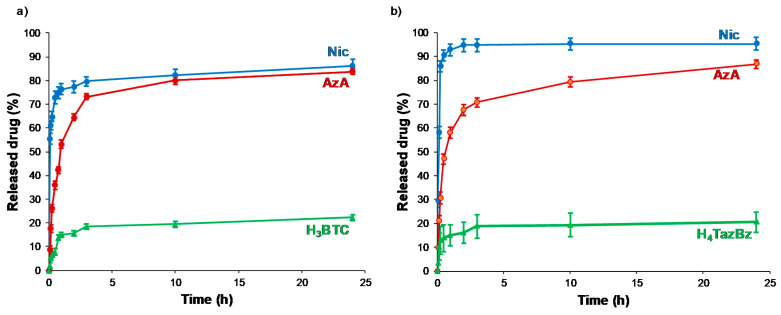
Nic and AzA co-release from (**a**) MIL-100 and (**b**) MIL-127. The leached (H_3_BTC and H_4_TazBz) ligands were also represented.

The ability to deliver the combined active cargoes from MOFs was investigated in order to consider them as topical drug delivery systems (DDS). Thus, mimicking the hydration of a cutaneous patch, the delivery of AzA and Nic was performed in dH_2_O at 32 °C under continuous stirring. The release kinetics were determined by quantifying the amount of the delivered drugs in the medium by HPLC as a function of time (Figure 4, SI S4). According to the drug-release profiles, an almost complete release of both drugs is achieved after 24 h. A fast release in the first 1 or 3 h for Nic and AzA was observed, respectively. The faster release of Nic, reaching a plateau after 2–3 h, could be explained by its higher hydrophilic character in comparison with the AzA drug. Then, a slow progressive release of more hydrophobic AzA is observed in the following 24 h. Resulting release kinetics seem to be well-adapted to the cutaneous delivery of AzA and Nic since topical formulations are usually applied for a maximum of 8–24 h period [64,65,66]. Further, it should be noted that, the faster kinetics of the Nic compared to AzA is beneficial because of the anti-inflammatory effects of Nic which reduce the possible skin irritations caused by AzA, as it was previously mentioned in the introduction.

The possible clinical translation of these MOFs@drugs systems as cutaneous DDS requires considering the total time and the total amount of released drug. So, besides being biocompatible, MIL-100 and MIL-127 materials are good candidates as cutaneous DDS. After 24 h, the total amount of released drugs is 0.19 g of Nic and 0.1 g of AzA *per* gram of MIL-100, and 0.19 g of Nic and 0.08 g of AzA per gram of MIL-127. The release profiles in play here lie within the same range when further compared to previous delivery studies on aspirin and ibuprofen in water for 1 day from MIL-100 (99 ± 1 and 84 ± 5%), MIL-127 (37 ± 2 and 48 ± 2%), and UiO-66 (96 ± 2 and 46 ± 2%, respectively) [57]. There are further drug delivery studies using MIL-100 and MIL-127 as delivery agents, however most of these studies are performed under different conditions (e.g., simulated gastric conditions, pH = 1.2) or intravenous conditions (e.g., phosphate buffer saline, simulated body fluid), hindering a direct comparison since it is known that the composition of the media (e.g., ions, proteins) strongly affects the resulting drug release kinetics.

To shed some light on the release kinetics and to gain further understanding on the involved mechanisms, short times of delivery (up to 1 h) were fitted to two different mathematical models (Figure S11, Table S4). AzA release from both MOFs seems to follow a zero-order kinetic (Equation (2)):

*Q_t_* = *K t*, (2)

where *Q_t_* indicates the concentration of released drug (mg·g^−1^), *t* is the time (*h*), and *K* is the kinetic constant (g·mg^−1^·h^−1^). This model is often used to describe the drug dissolution of several types of modified release pharmaceutical dosage forms, as well as matrix with low soluble drugs, and has been extensively used to described the drug release from porous materials [67]. The release process takes place at a constant rate, regardless of the remaining drug concentration.

On the other hand, the Nic release fits better to the Higuchi model (Equation (3)) [68]:

Q_t_ = *K_H_ t*^1/2^, (3)

where *Q_t_* indicates the concentration of released drug (mg·g^−1^), *t* is the time (h), and *K_H_* is the Higuchi kinetic constant (g·mg^−1^·h^−1/2^). In particular, the Higuchi model, which defines the short time behavior of the release of a dispersed drug from a homogeneous matrix, has been also applied to describe the diffusion of drugs from porous materials. This commonly used equation perfectly describes release processes in which the drug is dispersed in stable monolithic systems (in absence of changes during the release process), being the release purely controlled by diffusion [69]. In this sense, to confirm the integrity of the MOF matrixes during the release process, structural and chemical stability of both MOFs was confirmed by XRPD (Figure S12) and HPLC (monitoring the leaching of the constitutive ligands (<10% degradation; Figure 4). Further, considering that the external diffusion process around the MOF particles is minimized by a continuous stirring during the delivery assay, the desorption process might be only due to the drug movements through the pores of the frameworks. Except for the Nic release from NicAzA@MIL-100, with a regression factor (R^2^) value slightly below 0.99, the rest of the drugs releases can be empirically adjusted in the first hour to the Higuchi and zero-order models with R^2^ > 0.99. By estimating the *K* values, we confirmed that the Nic release is five times faster from MIL-100 than from MIL-127, suggesting an improved drug–MOF affinity. In contrast, the AzA kinetics of release is similar in both MOFs. Therefore, it can be assumed that MIL-127 leads to a well-controlled drug release, which might be associated to a good control of drug concentration and higher efficacy.

**Figure 5:**
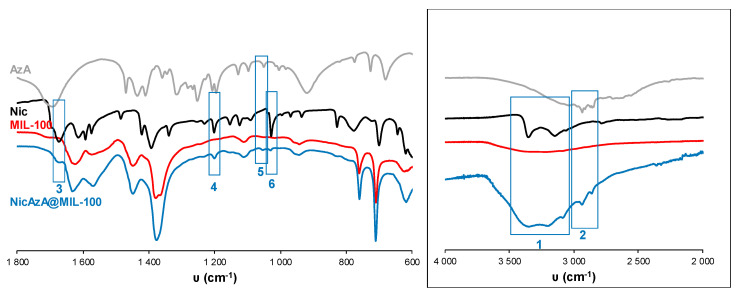
FT-IR spectra of empty MIL-100 and drugs-loaded NicAzA@MIL-100. The spectra of free drugs (Nic and AzA) have been included for comparison. FT-IR spectroscopic analysis clearly showed the presence of Nic and AzA into the NicAzA@MIL-100 loaded matrix: Nic δ(N-H) (3), Nic ν(C-NH_2_) (4), AzA ν(C-O) (5), and Nic δ(C-H) (6) bands. The inset shows a spectra magnification with the Nic ν(N-H) (1) and AzA ν(C-H) – ν(OH) (2) bands [70,71,72].

**Figure 6:**
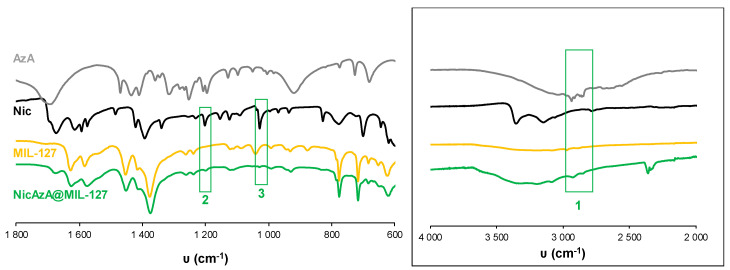
FT-IR spectra of empty MIL-127 and drugs-loaded NicAzA@MIL-127. The spectra of free Nic and AzA have been included for comparison. FT-IR spectroscopic analysis clearly showed the presence of Nic and AzA into the NicAzA@MIL-127 loaded matrix: Nic ν(C-NH_2_) (2), Nic δ(C-H) (3) bands. The inset shows a spectra magnification with the AzA ν(C-H) – ν(OH) (1) band [70,71,72].

In an attempt to rationalize the drug kinetics, apart from the drug diffusion through the pores and the hydrophilic/hydrophobic balance of the drug and MOF, one could expect that drug–matrix interactions could also contribute to the resulting release profiles. Considering the presence of reactive functional groups in the drugs structure (amide, carboxylate, pyridine), the formation of specific host–guest interactions was investigated by Fourier transform infrared spectroscopy (FTIR). The FTIR spectroscopic analysis clearly shows the presence of the main bands corresponding to both drugs in the loaded materials (see details in Figure 5 and Figure 6). Remarkably, the absence of the C=O vibrational band at ca. 1691 cm^−1^, corresponding to the free carboxylic group in the free AzA [70], is consistent with the encapsulation of the sodium salt and suggests the potential coordination of this group with the coordinatively unsaturated metal sites (CUS) of the MIL-100 and MIL-127. The formation of such an interaction could also contribute to the slower release of AzA when compared with Nic. Further, FTIR spectrum of NicAzA@MIL-100 confirmed a shift in the wavelengths in comparison with the pure Nic characteristic bands: N-H (from 3350 to 3353 cm^−1^) and C-N (from 1201 to 1199 cm^−1^) [71], respectively, suggesting the interaction between drug moieties and the MIL-100 framework. In addition, one could tentatively propose the formation of hydrogen bonds between these N-H or C-N groups, and the hydroxyl groups present within the framework. A similar shift of the C-N band is also observed in NicAzA@MIL-127 (from 1201 to 1199 cm^−1^). As a whole, one can conclude that the drug delivery is governed by the balance between the MOF–drug interaction, the drug diffusion through the pore, but also the degradation of the material.

As concluding remark, it should be noted here that it is of great importance to know the drug delivery performance using real skin conditions, which includes water vapor instead of liquid solutions, and subsequently might make the drug delivery slower. The results obtained here may not fully or accurately predict the effects on skin, and therefore ex vivo tests using skin are mandatory to consider these systems as good candidates as cutaneous DDS.

### 3.3. Skin Permeation

Topically applied products may target different sites in one or more skin layers (i.e., epidermis, dermis and hypodermis), skin appendages (e.g., hair follicles with associated sebaceous glands, sweat glands and nails), and underlying tissues [73]. Particularly, anti-acne APIs need to cross the *stratum corneum* barrier and access the epidermal and dermal sites, mainly the epidermal–dermal junction and hair follicles, in sufficient quantity to exert a therapeutic effect [74,75].

The high total drugs encapsulation rate (total 77.4 and 48.1 wt.% for MIL-100 and MIL-127, respectively) and their kinetics of release in water (between 95 to 84% after 1 day) encourage us to investigate the Nic and AzA permeation through the skin. Prior to the evaluation of the drug-loaded MOFs in terms of APIs delivery under simulated real conditions, composite patches well-adapted for cutaneous administration were prepared. The patches based on the drug-containing MOFs and a polymer were shaped by an easy and rapid press-molding approach consisting of three simple steps: (i) a component milling, (ii) a mixing step, and (iii) the preparation of a wafer with the resulting mixture by applying uniaxial pressure. In order to provide patches with suitable topical properties such as biocompatibility, prophylaxis against infections, lubricity, cell/microorganism adhesion or susceptibility to an external stimulus (pH, temperature, ionic strength, etc.,) [76,77,78], low molecular weight polyvinyl alcohol (PVA) was selected. Aside from its biocompatibility, PVA shows good mechanical strength and thermal stability, excellent film-formation qualities, low cost, and commercial availability. After the press-molding, four types of patches (see compositions in Table S5) of 12.0 ± 0.1 mm diameter and 0.6 ± 0.1 mm thickness were obtained: the drugs-loaded MOFs with PVA (denoted as NicAzA@MIL-100_patch and NicAzA@MIL-127_patch) and the PVA containing the same drugs content, used as controls (called NicAzA@A, with the same drugs content as MIL-100; and NicAzA@B, with the same drugs content as the MIL-127). The maintenance of the crystalline structure of MIL-100 and MIL-127 solids upon the patches processing was confirmed by XRPD (Figure S13).

**Figure 7:**
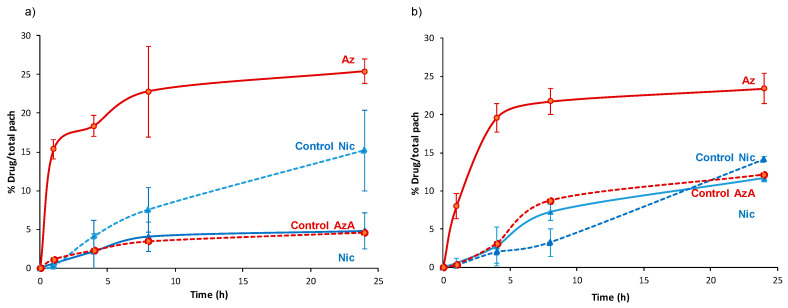
Nic and AzA delivery profile from (**a**) NicAzA@MIL-100_pach, and (**b**) NicAzA@MIL-127_pach to the receptor compartment of the Franz chamber (diffused Nic and AzA).

**Figure 8:**
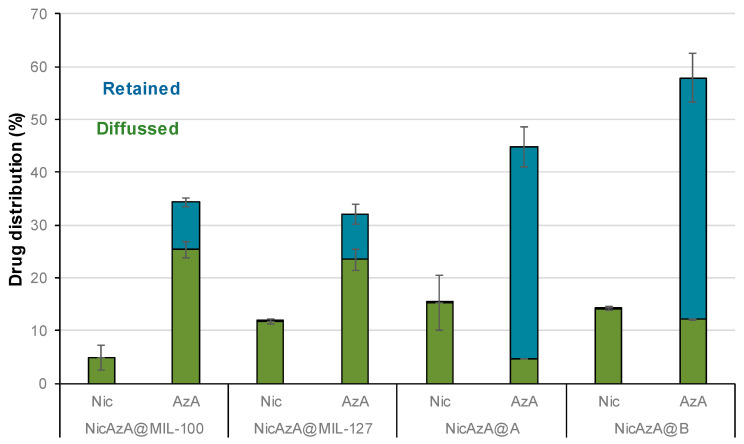
Skin distribution of Nic and AzA (%) after 24 h of ex vivo permeation tests.

Then, ex vivo permeation assays were carried out using Franz diffusion chambers, accepted as a good model for the determination of the release and skin barrier crossing of APIs (Figure 7) [79]. They consist of two compartments (receptor and donor) separated by a skin membrane (the inner region of the porcine ear as a suitable model of human skin permeability; see Materials and methods section). The *stratum corneum* of the skin was placed in the donor compartment in contact with the patch formulations covered by an impermeable film (Leukoflex^®^) to avoid dryness, and the receptor chamber was filled with a phosphate-buffer solution (PBS, pH = 7.4). Porcine skin is generally preferred because of its structural similarity to the human skin in terms of: hair growth density (~20 hairs·cm^−2^) and the presence of structures such as Langerhans cells and rete ridges, *stratum corneum* thickness and contents such as glycosphingolipids and ceramides, stratified, multi-layered, keratinizing epithelium, thickness of the viable epidermis (~70 µm), and collagen fibers arrangement in the dermis [80]. The porcine skin was previously dermatomed to a 1.2 mm-thickness, removing the fatty layer and keeping the upper layers. Considering the latter, the amount of drugs able to reach the adipose tissue and/or systemic circulation could be easily quantified by HPLC within the receptor chamber (referred as diffused Nic or AzA and expressed as the % with respect to the total drugs cargo, Figure 8). In addition, the drugs content retained within the skin was also determined (named as retained Nic or AzA).

First, we should mention that during 24 h in contact with the patches, we observe an intact skin without irritation, corrosion, or reddening, suggesting the good biocompatibility of the Nic and AzA-containing composite patches. These safety tests were also confirmed with human volunteers, showing no irritation, itching sensation, warm, or humidity after the exposure of the pellets after 8 h (Supplementary Materials Figure S16). A progressive skin bypass of both drugs was evidenced, being more important for the AzA than Nic. After a typical cutaneous contact time (8 h), Nic progressively bypasses the skin barrier from both MOF systems (4.1 ± 1.9% or 1.14 mg·cm^−2^ for NicAzA@MIL-100_pach, and 7.2 ± 1.0% or 0.58 mg·cm^−2^ for NiAzA@MIL-127), being comparable with other studied formulations (e.g., 5.1% or 5.3 ± 1.5 ug·cm^−2^ in Olay total effects, 4.4% or 15 ± 3.4 ug·cm^−2^ in dimethyl isosorbide, 3.3% or 1.8 ± 0.4 µg·cm^−2^ in polyethylene glycol, data corresponding to a 24 h period in human tissue) [81]. Similar diffusion profiles are observed for the MOF-based patches and the controls, probably associated with the fact that Nic is rapidly released from the MOFs (~1 h; Figure 4) and it is readily absorbed by the skin [82]. In fact, Nic is a precursor to the cofactors nicotinamide adenine dinucleotide (NADH) and nicotinamide adenine dinucleotide phosphate (NADPH), promoting their increase upon Nic topical administration. NADPH is a cofactor for synthesis of fatty acids and lipids such as ceramides, which explains why topical application of Nic enhances skin barrier lipids and modifies barrier permeation [83].

Regarding AzA, an important amount is able to diffuse through the skin (23 ± 6% or 2.5 mg·cm^−2^ for NicAzA@MIL-100, and 22 ± 1.7% or 1.75 mg·cm^−2^ for NicAzA@MIL-127 after 8 h), being significantly higher than the AzA skin diffusion from the PVA-based controls (3.5 ± 0.18% or 0.42 mg·cm^−2^, and 8.7 ± 0% or 0.86 mg·cm^−2^ for A and B, respectively).

The improved skin bypass might be related with the formation of AzA ionized species, since the PVA controls are formulated using the commercially used acid form of AzA, but MOFs with the ionized AzA, which is known to enhance its skin permeation in comparison with its acid counterpart [44]. Further, the diffused AzA values obtained here are 30-times better than the reported ones (~4% for commercialized products 73 µg·cm^−2^, data corresponding to 24 h in porcine tissue) [15]. Considering the combined drug release, NicAzA@MIL-100_pach is able to deliver 1.14 mg·cm^−2^ of Nic and 2.5 mg·cm^−2^ of AzA, and NicAzA@MIL-127_pach can release 0.58 mg·cm^−2^ of Nic and 1.75 mg·cm^−2^ of AzA in 8 h, one could suggest that the formulation based on MIL-100 is more effective.

Despite a proper drugs skin crossing of the tested patch formulations, an important amount of the initially loaded drugs did not reach the receptor compartment. In this regard, the amounts of drugs retained within the skin were estimated as the sum of the amounts of the *stratum corneum* and viable skin layered (epidermis and dermis; see Figure 8). Regarding Nic, as it was previously mentioned, it suffers from poor skin retention (~1% of the applied dose after 72 h in human skin) [19], being here within the range of other tested formulations with proved efficiency in the recession of inflammatory reaction (from 1.89 to 0 mg vs. from 0.66 to 0 mg in a Nic-based gel) [20]. On the other hand, the AzA skin retention from the MOF-based patches (8.89% or 0.34 mg, and 8.58% or 0.27 mg for NicAzA@MIL-100_pach and NicAzA@MIL-127_pach, respectively) is similar or even improved when compared to other reported formulations (e.g., 3.41% or 0.13 mg of AzA in a commercial cream, 4.74% or 0.40 mg of AzA in an ethanolic-based gel, and 11.87% or 0.58 mg of AzA in a microemulsion) [15]. Notably, the AzA retained within the skin in the control formulations was significantly higher than that in the MOF-patches, more precisely around 4.5 and 5.5-fold higher for MIL-100 and MIL-127, respectively. This result highlights the influence of the formulation on the drug biodistribution and bioavailability and could be explained by the fact that the total drugs content is available in the polymer from the beginning, while the AzA is progressively released from the MOF to the PVA in the composite patches. Note here that the higher permeation of AzA from MOF-based patches could also allow transdermal drug therapies, recently been proposed for acne vulgaris treatment [84]. Note here that the retention of AzA within the skin could improve the anti-acne efficiency of the formulation.

Finally, the diffusion flux (*J*) was estimated for each formulation at 24 h (see details in SI, Table S6). The diffusion flux (*J*) for Nic was 4 and 1.5-fold faster from the controls than for NicAzA@MIL-100 and NicAzA@MIL-127, respectively, indicating the diffusion control by the progressive drug release and extending the half-life of this drug. In contrast, AzA diffusion flux after 4 h was 4 and 5.5-fold times slower from the controls than from the NicAzA@MIL-127 or NicAzA@MIL-100, respectively. This could be related with the fact that the MOFs release the ionized AzA form (sodium salt) with an improved skin permeation, while the PVA controls are formulated using the acid AzA form with lower permeation. In fact, it is well-known that the formulation strongly impacts the cutaneous permeation of APIs [85]. Comparing with commercial formulations, a more controlled diffusion of Nic is obtained here (e.g., 45.07 µg·cm^−2^·h^−1^ (Nic-2-pyrrolidone), and 8.97 µg·cm^−2^·h^−1^ (Nic-dimethyl isosorbide/Miglyol 812N^®^) [86], whereas AzA the obtained diffusion flux is within the range of commercial devices (e.g., 6.70 µg·cm^−2^·h^−1^ (AzA-EtOH/pH 7 buffer) [87], and 80.5 µg·cm^−2^·h^−1^ (AzA-FINACEA^®^)) [15]. Altogether, these findings suggest that these NicAzA-containing MOF-based patches can efficiently deliver both drugs to the skin in active concentrations.

## 4. Conclusions

In the present work, we have proposed two novel combined anti-acne topical formulations based on biocompatible Fe-MOF composite patches. The co-encapsulation of the anti-inflammatory Nic and antibiotic AzA inside the pores of MIL-100 and MIL-127 was successfully achieved using a simple, biofriendly, and efficient impregnation method, reaching exceptional cargoes (77.4 and 48.1 wt.%). Under simulated skin conditions, the active cargoes were progressively released within a duration compatible with topical therapies (8–24 h), with AzA delivery being faster than that of Nic. Further, composite patches, suitable for cutaneous administration, were prepared based on the drug co-loaded MOFs and the biocompatible polymer PVA by a simple and rapid press-molding approach. These cutaneous PVA-MOF formulations maintain the high drug-loading capacity (with a total drug content of 38.7 and 24.1 wt.% for MIL-100 and MIL-127, respectively), while allowing a controlled delivery of their cargoes, permeating through the skin to the active target sites. Interestingly, the total amount of drug retained or diffused through the skin is within the range (Nic), or even better (AzA) than commercial formulations.

## Figures and Tables

**Table 1:** Nic and/or AzA encapsulation loading, encapsulation efficiency (*EE*), and variation in BET surface areas (S_BET_) and pore volume (V_p_) after drug encapsulation in MIL-100 and MIL-127 Fe-based MOFs.

		Encapsulation Rate Mol·mol^−1^ (wt.%)		*EE* (%)		Porosity Variation Before and After Encapsulation	
		Nic	AzA *	Nic	AzA	ΔS_BET_ (m^2^·g^−1^)	ΔV_p_ (cm^3^·g^−1^)
**MIL-100**	**C**	2.87 (58.4 ± 1.4)	0.65 (19 ± 3)	17.3	46.5	2080	0.88
	**S**	1.00 (15.8 ± 1.5)	0.23 (9.1 ± 0.2)	5.8	21.2	-	-
**MIL-127**	**C**	2.23 (34.1 ± 0.9)	0.57 (14 ± 4)	10.6	35.0	970	0.34
	**S**	0.9 (16.0 ± 0.3)	2.1 (34.3 ± 1)	7.35	83.7	-	-

C = combined encapsulation; S = single encapsulation. * Note here the encapsulation of the disodium salt (see SI).

## Supplementary Materials

The following are available online at https://www.mdpi.com/2079-4991/10/12/2296/s1. Figure S1: Calibration plot by HPLC method, UV-vis spectra and chromatogram of H_3_BTC; Figure S2: Calibration plot by HPLC method, UV-vis spectra and chromatogram of H_4_TazBz, Figure S3: Calibration plot by HPLC method, UV-vis spectra and chromatogram of der-AzA, Figure S4: Calibration plot by HPLC method, UV-vis spectra and chromatogram of Nic, Figure S5: TGA of NicAzA@MIL-100, Figure S6: TGA of NicAzA@MIL-127, Figure S7: N_2_ sorption studies, Figure S8: XRPD of one drug loaded materials, Figure S9: TGA of one drug loaded materials, Figure S10: FT-IR spectra of one drug loaded materials, Figure S11: Fitting of Nic and AzA delivery data, Figure S12: XRPD patters after drugs release process, Figure S13: XRPD patters of the prepared patches, Figure S14: Skin irritation test images, Table S1: Composition of the drugs loaded MOFs, Table S2: DLS characterization of pristine and drug loaded MOFs, Table S3: BET surface area and pore volume comparison, Table S4: Release kinetics, Table S5: composition of composite patches, Table S6: Diffusion flux data.
