# Supplementary material for: Combined Cutaneous Therapy Using Biocompatible Metal-Organic Frameworks
Source: Nanomaterials (Basel). 2020 Nov 25;10(12):2296. doi: 10.3390/nano10122296 (PMC7760737; doi:10.3390/nano10122296)
Supplement: Supplementary file 1 [file nanomaterials-10-02296-s001.pdf]

nanomaterials

Supporting Information

Combined Cutaneous Therapy Using Biocompatible Metal-Organic Frameworks

Seyed Dariush Taherzade [1,2,†], Sara Rojas [1,†], Janet Soleimannejad [2] and Patricia Horcajada [1,*]

1 Advanced Porous Materials Unit (APMU), IMDEA Energy, Av. Ramón de la Sagra 3, 28935 Móstoles-Madrid, Spain; d.taherzade@ut.ac.ir (S.D.T.); sara.rojas@imdea.org (S.R.)
2 School of Chemistry, College of Science, University of Tehran, P.O. Box 14155-6455, 1417614411, Tehran, Iran; janet_soleimannejad@khayam.ut.ac.ir
* Correspondence: patricia.horcajada@imdea.org; Tel.: +(34)-91-737-11-20
† These authors contributed equally to this work.

Table of Content

S1. High Performance Liquid Chromatography (HPLC) conditions S3

S2. Drugs encapsulation studies S5

S3. NicAzA@Fe-MOF characterization S6

S4. Drugs delivery studies S10

S5. Nic and AzA skin permeation test S11

S6. References S13

S1. High Performance Liquid Chromatography (HPLC) measurement conditions

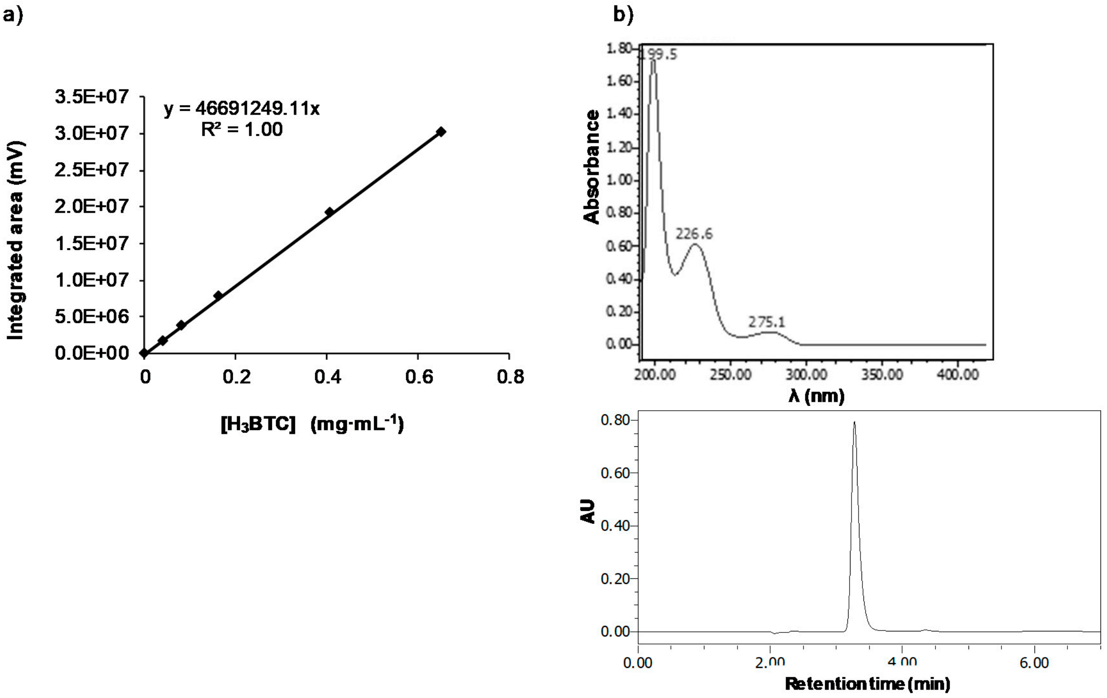

Figure S1. (**a**) Standard calibration plot of H_3BTC by HPLC method, and (**b**) UV-vis spectra and chromatogram of H_3BTC.

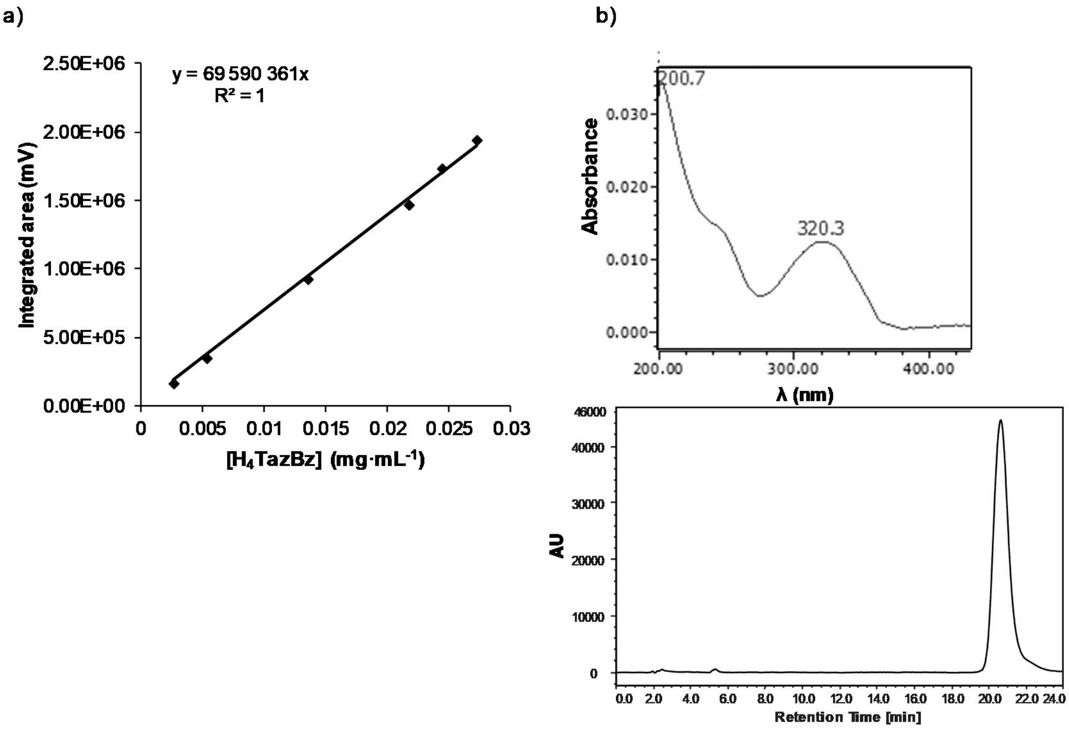

Figure S2. (**a**) Calibration plot of H_4TazBz by HPLC method, and (**b**) UV-vis spectra and chromatogram of H_4TazBz.

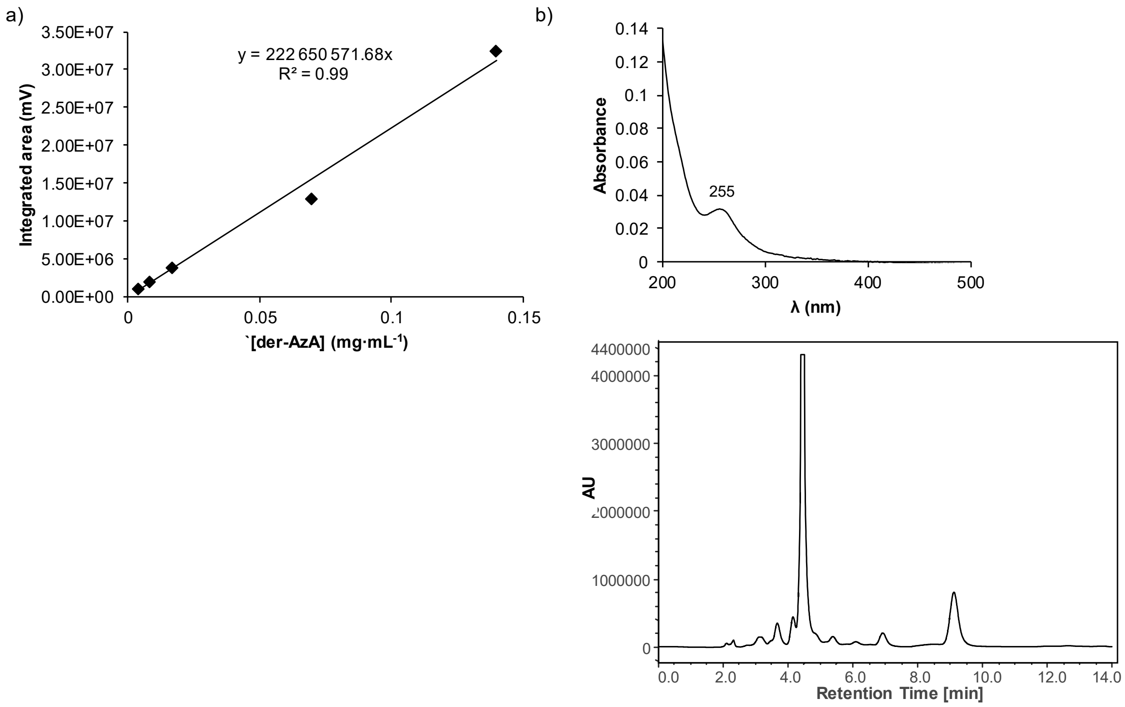

Figure S3. (**a**) Calibration plot of der-AzA by HPLC method, and (**b**) UV-vis spectra and chromatogram of der-AzA.

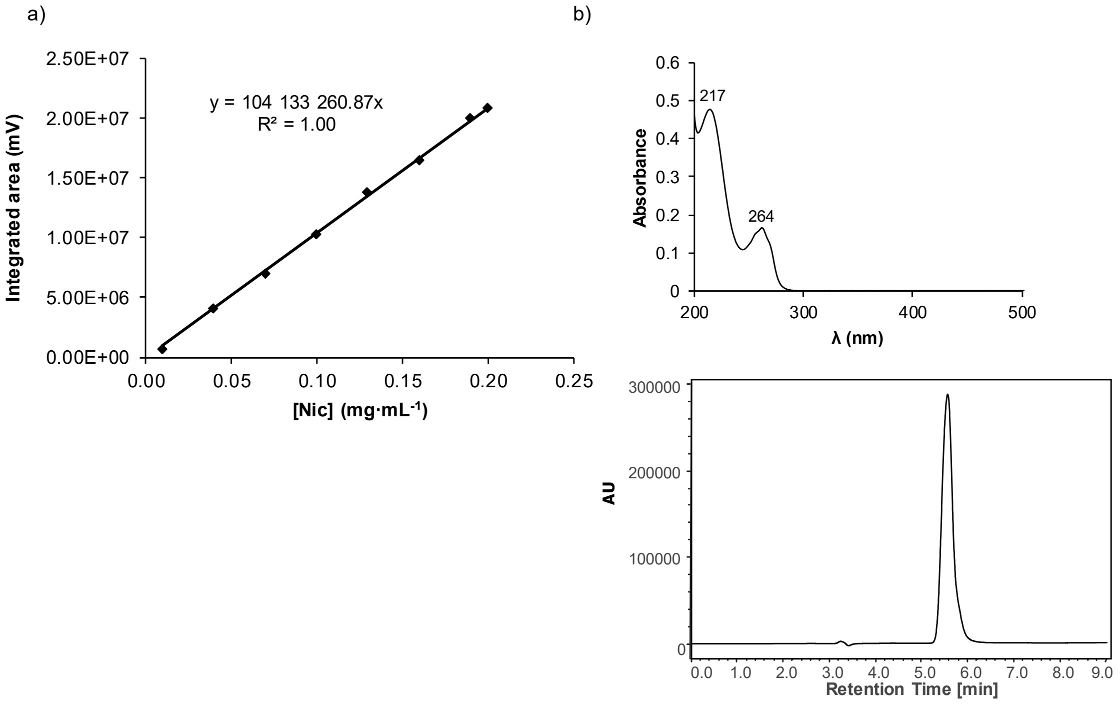

Figure S4. (**a**) Calibration plot of Nic by HPLC method, and (**b**) UV-vis spectra and chromatogram of Nic.

S2. Drugs encapsulation studies

Table S1. Composition of the drugs (C: combined, S: single) loaded MOFs, with the total drug content (mol·mol^{-1} and wt.%) and leached ligand (mol·mol^{-1} and wt.%; H_3BTC or H_4TazBz) quantified by HPLC, EA and TGA; and encapsulation efficiently (%, EE) during the encapsulation process (%).

		**HPLC mol·mol^{-1} (wt%)**			**TGA mol·mol^{-1} (wt%)**		**EA mol·mol^{-1} (wt%)**		**EE (%)**	
		**Nic**	**AzA**	**Ligand**	**Nic**	**AzA**	**Nic**	**AzA**	**Nic**	**AzA**
**MIL-100**	C	2.87 ± 0.04 (58.4 ± 1.4)	0.65 ± 0.02 (19.1 ± 3.1)	0.11 ± 0.00 (3.6 ± 1.8)	2.96 (60.1)	0.65 (19.1)	2.40 ± 0.04 (51.7 ± 1.6)	0.65 ± 0.02 (17.5 ± 3.1)	17.3	46.5
	S	-	0.23 ± 0.01 (9.1 ± 0.2)	0.09 ± 0.00 (3.0 ± 1.1)	1.00 (15.8)	0.35 (8.6)	-	-	5.8	21.2
**MIL-127**	C	2.23 ± 0.02 (34.1 ± 0.9)	0.57 ± 0.02 (14.2 ± 3.5)	0.17 ± 0.00 (8.0 ± 2.3)	2.28 (35.3)	0.57 (14.2)	2.28 ± 0.04 (28.1 ± 1.8)	0.96 ± 0.02 (14.6 ± 2.1)	10.6	35.0
	S	0.90 ± 0.02 (16.0 ± 0.3)	-	0.14 ± 0.02 (6.0 ± 1.3)	0.89 (15.7)	2.1 (34.3)	-	-	7.35	83.7

Proposed formulas with combined composition

NicAzA@MIL-100
Elemen. Anal. Calc. (%) for:
$[Fe_3O(OH)(H_2O)_2(C_6H_3(CO_2)_3)_2]\cdot(H_2O)_{11}(C_9H_{14}Na_2O_4)_{0.65}(C_6H_6N_2O)_{2.4}$
(MW: 1292.99 g·mol^{-1}). C(35%), H(3.34%), N(5%); found: C(37%), H(2.23%), N(5.37%)
Residue after thermal treatment: $(Na_2O)_{0.65}(Fe_2O_3)_{1.5}$; calculated: 21.6%; found: 25.75%

NicAzA@MIL-127
Elemen. Anal. Calc. (%) for:
$[Fe_3O(OH)_{0.88}Cl_{0.12}(C_{16}N_2O_8H_6)_{1.5}]\cdot(H_2O)_{14}(C_9H_{14}Na_2O_4)_{0.57}(C_6H_6N_2O)_{2.28}$
(MW=: 1397.10 g·mol^{-1}). C(39%), H(2.8%), N(8.4%); found: C(41.28%), H(2.79%), N(8.92%)
Residue after thermal treatment: $(Na_2O)_{0.57}(Fe_2O_3)_{1.5}$; calculated: 13.6%; found: 11.57%

S3. NicAzA@Fe-MOF characterization

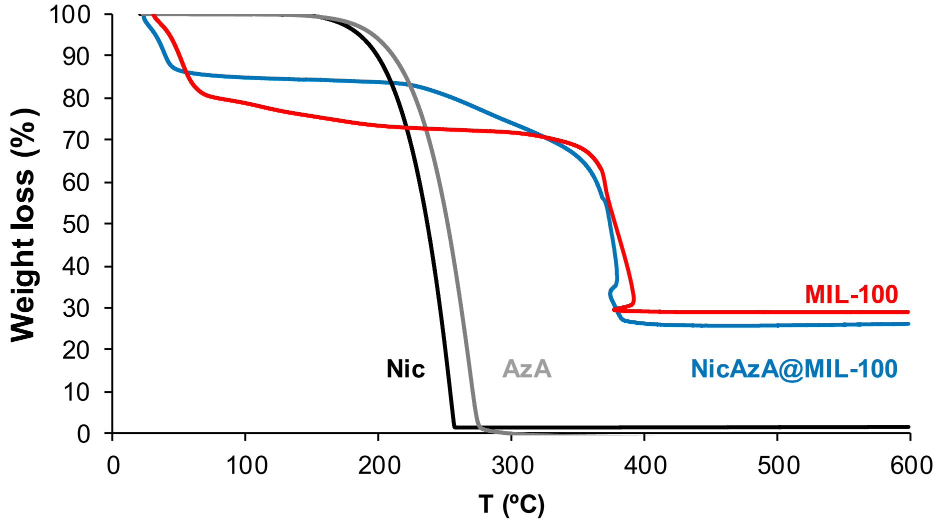

Figure S5. TGA for MIL-100, NicAzA@MIL-100, Nic and AzA.

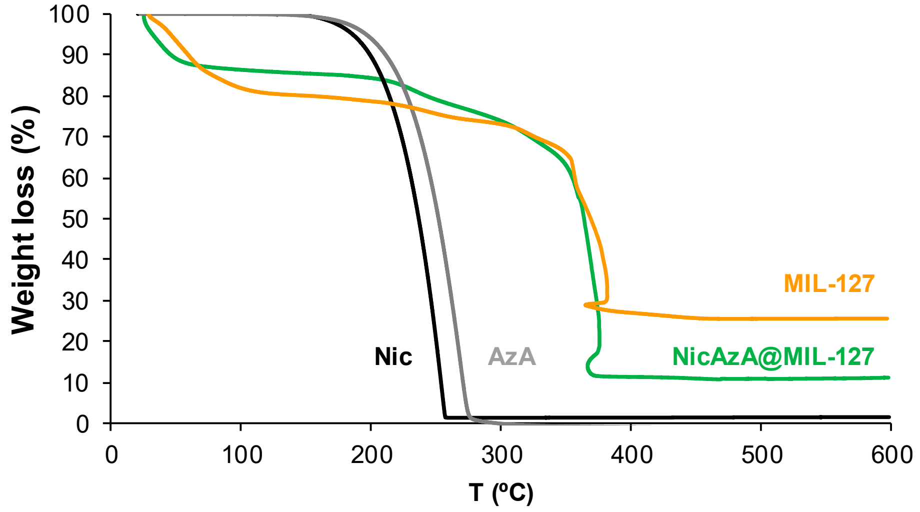

Figure S6. TGA for MIL-127, NicAzA@MIL-127, Nic and AzA.

Table S2. Size and polydispersity index (PDI) measured in 5 min-aged suspensions in water to ensure comparable situation. Note the high dispersity of the samples PDI.

	MIL-100	NicAzA@MIL-100	MIL-127	NicAzA@MIL-100
**Size (nm)**	1284 ± 1639	901 ± 332	351 ± 42	394 ± 85

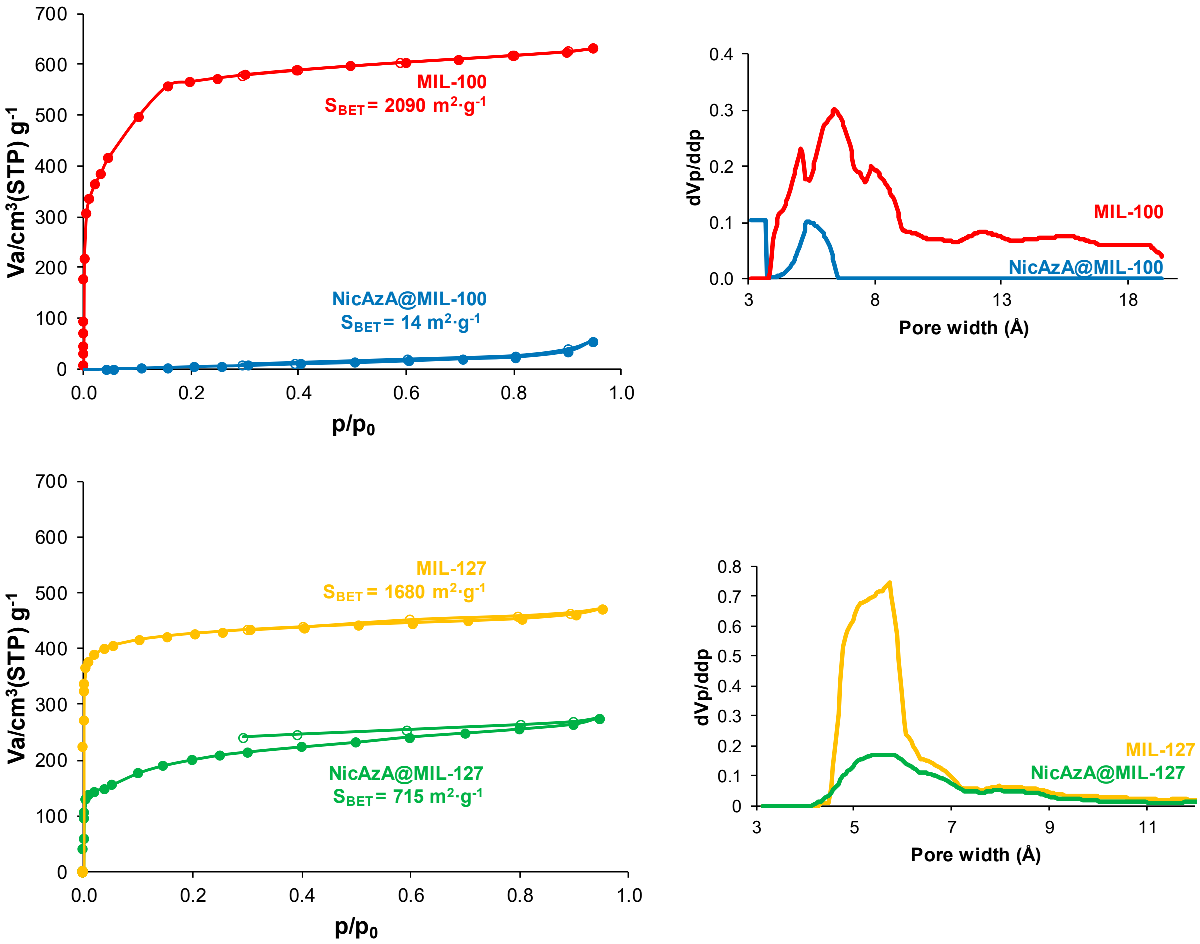

Figure S7. N_2 sorption isotherms at 77 K for empty MIL-100 and MIL-127 and its corresponding drugs loaded materials NicAzA@MIL-100 and NicAzA@MIL-127. Empty symbols correspond to the desorption branch. Horvath-Kawazoe (HK) method was used to determinate the pore size distribution. The incorporation of drugs is accompanied by a dramatic reduction of the N_2 sorption capacity of both Fe-MOFs. The drugs adsorption leads to a large reduction in MIL-100 pore size accompanied with a reduction in pore volume (~ 99%), while in MIL-127 the pore size is maintained and there is a reduction in pore volume (~ 51%).

Table S3. BET surface area S_{BET} ($m^2 \cdot g^{-1}$) and total pore volume V_P (p/p_0=0.3, $cm^3 \cdot g^{-1}$) of MOFs before and after the drugs encapsulation process.

	S_{BET} ($m^2 \cdot g^{-1}$)	V_P
**MIL-100**	2090	0.89
**MIL-127**	1680	0.67
**NicAzA@MIL-100**	10	0.01
**NicAzA@MIL-127**	710	0.33

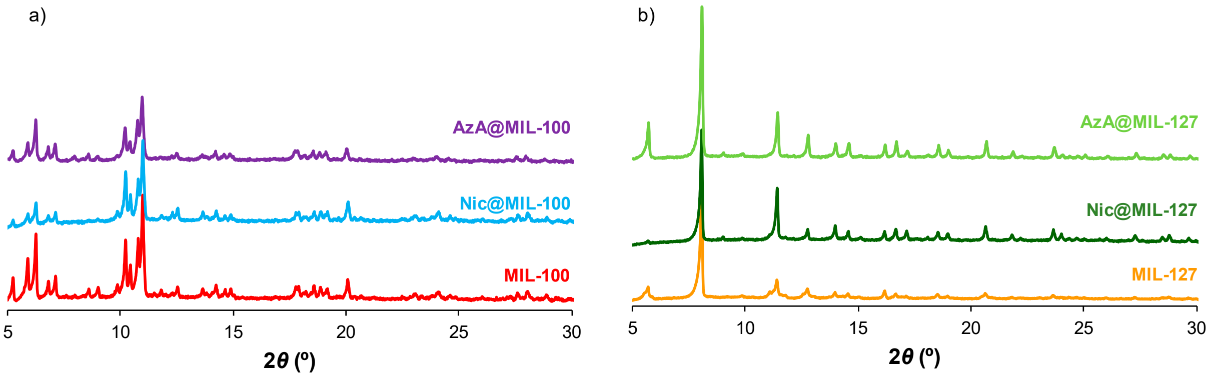

Figure S8. XRPD pattern of one drug loaded materials (a) AzA@MIL-100, Nic@MIL-100, and (b) AzA@MIL-127, Nic@MIL-127 compared with the pristine porous materials. X-ray powder diffraction (XRPD) patterns evidence that the drug-loading process does not alter the crystalline structure of the porous materials.

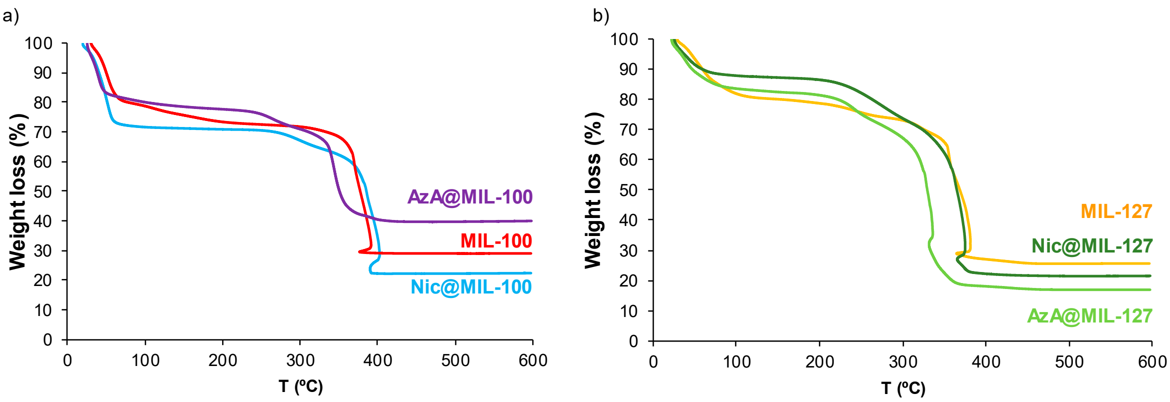

Figure S9. TGA of one drug loaded materials (a) AzA@MIL-100, Nic@MIL100, and (b) AzA@MIL-127, Nic@MIL-127 compared with the pristine porous materials.

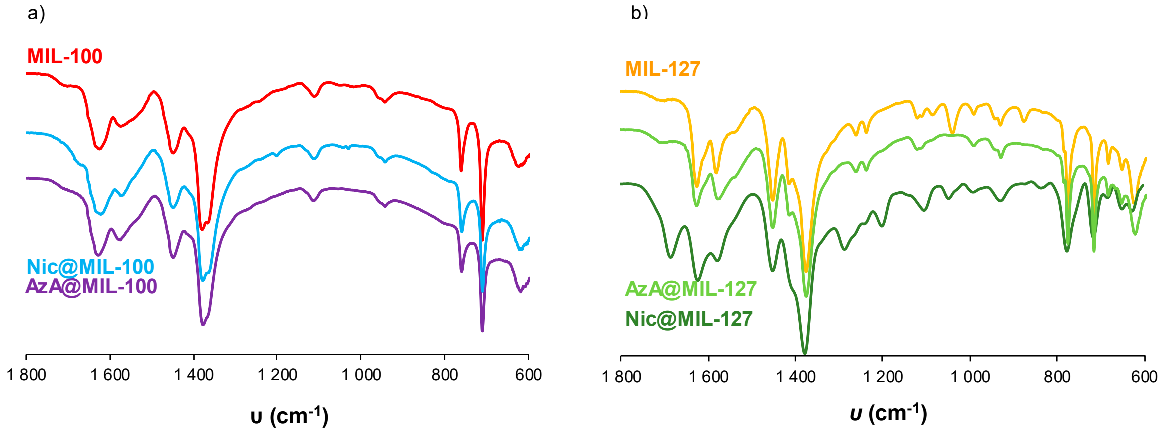

Figure S10. FT-IR spectra of one drug loaded materials (a) AzA@MIL-100, Nic@MIL100, and (b) AzA@MIL-127, Nic@MIL-127 compared with the pristine porous materials.

S4. Drugs delivery studies

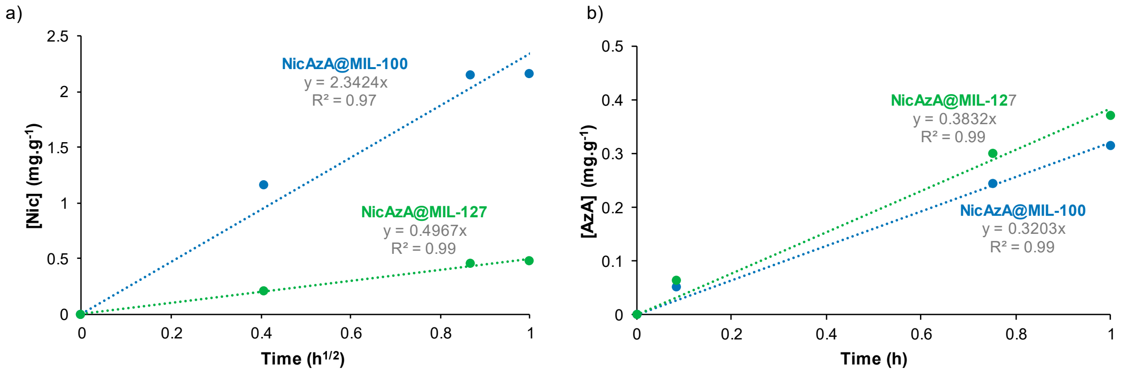

Figure S11. Fitting of the (**a**) Nic and (**b**) AzA delivery data from Fe-MOFs to a Higuchi model and a zero-order kinetic, respectively.

Table S4. Release kinetics including the kinetic model (Higuchi-H or Zero-O) used in the data fitting, kinetics constant of release (*K*, $mg{\cdot}g^{-1}{\cdot}h^{-1/2}$ or $mg{\cdot}g^{-1}{\cdot}h^{-1}$), total release time (h), and total drug release (%).

	Nic			AzA		
**Material**	**Kinetic model**	***K*** **($mg{\cdot}g^{-1}{\cdot}h^{-1/2}$)**	**Release time (h) and drug (%)**	**Kinetic model**	***K*** **($mg{\cdot}g^{-1}{\cdot}h^{-1}$)**	**Release time (h) and drug (%)**
**MIL-100**	H	2.34	1<br>90 ± 3	0	0.32	1<br>85 ± 2
**MIL-127**	H	0.50	1<br>99 ± 1	0	0.383	1<br>85 ± 3

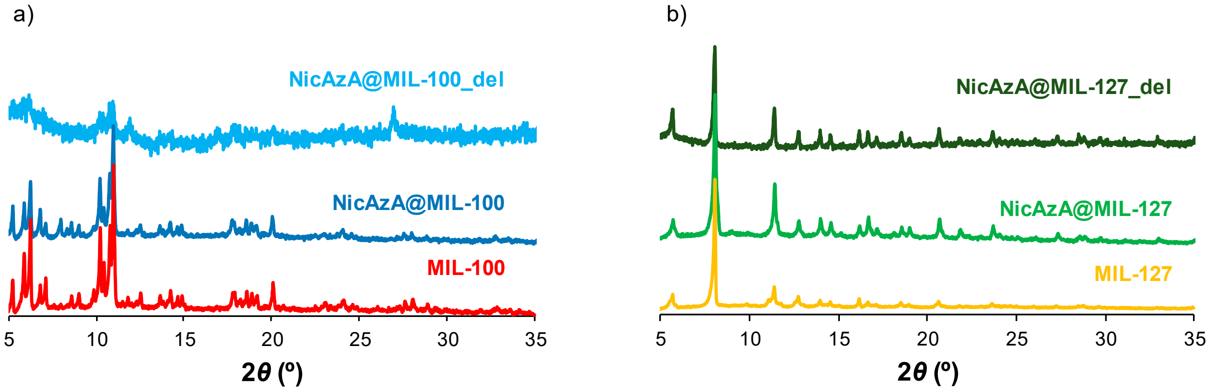

Figure S12. XRPD patterns of pristine MOFs ((**a**) MIL-100 and (**b**) MIL-127), their corresponding drugs loaded materials (NicAzA@MIL-100 and NicAzA@MIL-127), and the final solids obtained after drugs release process (NicAzA@MIL-100_del and NicAzA@MIL-127_del).

S5. Nic and AzA skin permeation test

Table S5. Composition of composite patches in mg.

Patch	Drug loaded@MOF (mg)	PVA (mg)	Drug (mg)
NicAzA@MIL-100_patch	50	50	-
NicAzA@MIL-127_patch	50	50	-
NicAzA@A (as in MIL-100)	-	81.72	4.73 (AzA)<br>13.55 (Nic)
NiAzA@B (as in MIL-127)	-	86.41	3.84 (AzA)<br>9.75 (Nic)

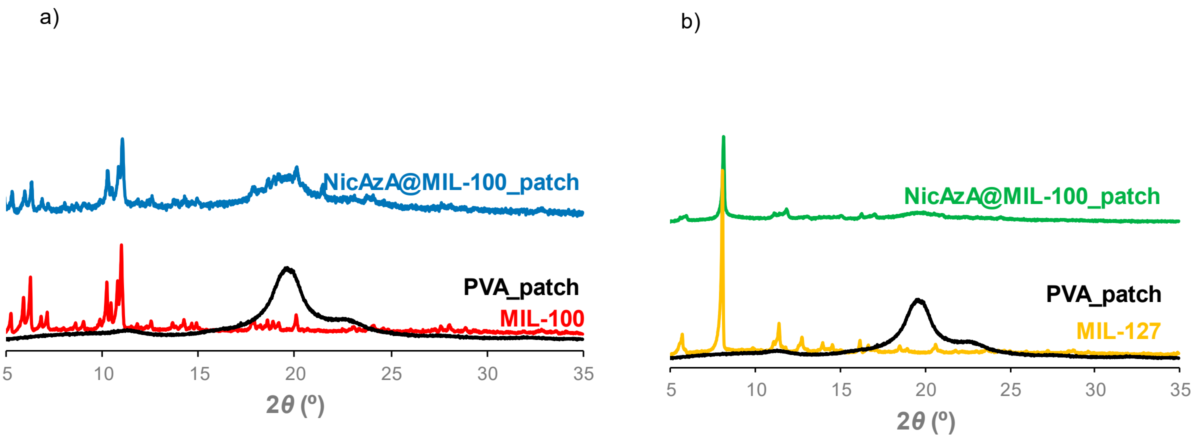

Figure S13. XRPD patterns of (a) NicAzA@MIL-100_patch and (b) NicAzA@MIL-127_patch, together with pristine MIL-100 and MIL-127, and PVP_patch.

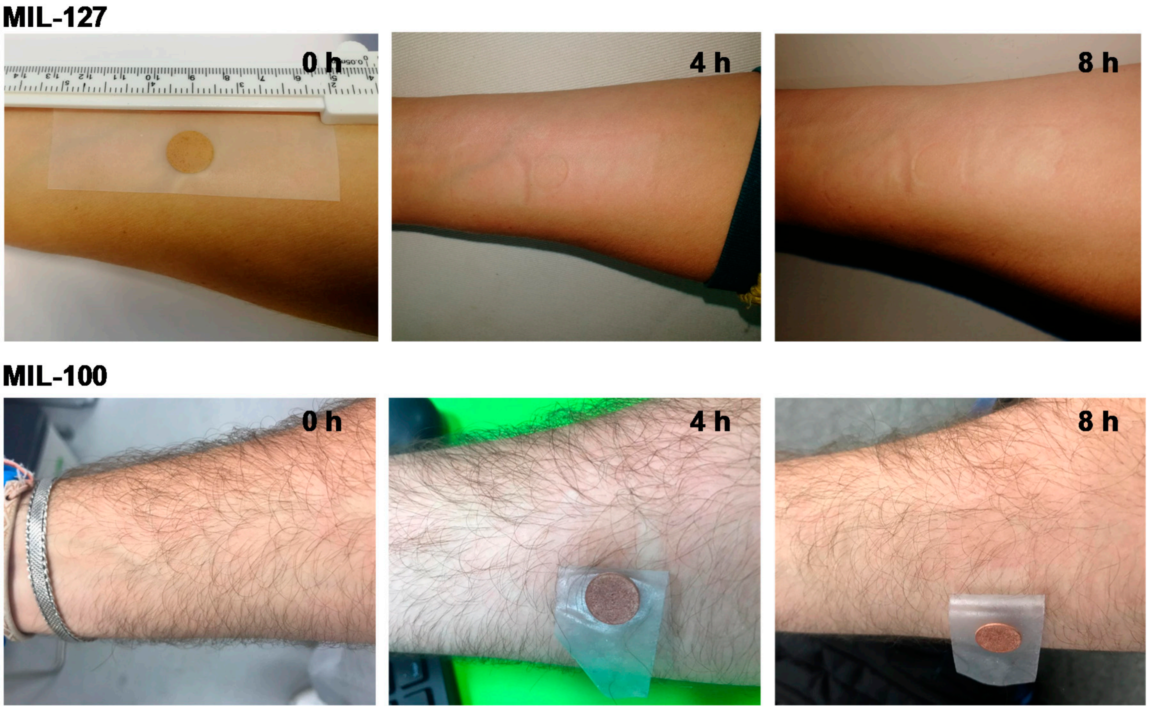

Figure S14. Skin irritation test for MIL-100_pach and MIL-127_pach. No irritation was observed in none of the volunteers.

Table S6. Diffusion flux (*J*, μg·cm^{-2}·h^{-1}) for all tested patches at 24 h.

**Time (h)**	***J* (μg·cm^{-2}·h^{-1})**	
	**24**	
	**Nic**	**AzA**
NicAzA@MIL-100	18.19	33.52
NicAzA@A	66.73	8.75
NicAzA@MIL-127	29.07	22.93
NicAzA@B	44.65	23.03

S6. References

1. Chevreau, H.; Permyakova, A.; Nouar, F.; Fabry, P.; Livage, C.; Ragon, F.; Garcia-Marquez, A.; Devic, T.; Steunou, N.; Serre, C.; et al. Synthesis of the biocompatible and highly stable MIL-127(Fe): From large scale synthesis to particle size control. *CrystEngComm* **2016**, *18*, 4094–4101, doi:10.1039/c5ce01864a.
2. Canioni, R.; Roch-Marchal, C.; Sécheresse, F.; Horcajada, P.; Serre, C.; Hardi-Dan, M.; Férey, G.; Grenèche, J.-M.; Lefebvre, F.; Chang, J.-S.; et al. Stable polyoxometalate insertion within the mesoporous metal organic framework MIL-100(Fe). *J. Mater. Chem.* **2011**, *21*, 1226–1233, doi:10.1039/c0jm02381g.
3. Capristo, E.; Mingrone, G.; De Gaetano, A.; Addolorato, G.; Greco, A. V.; Gasbarrini, G. A new HPLC method for the direct analysis of triglycerides of dicarboxylic acids in biological samples. *Clin. Chim. Acta* **1999**, *289*, 11–21, doi:10.1016/S0009-8981(99)00145-X.
4. Bretti, C.; Crea, F.; Foti, C.; Sammartano, S. Solubility and activity coefficients of acidic and basic nonelectrolytes in aqueous salt solutions. 2. Solubility and activity coefficients of suberic, azelaic, and sebacic acids in NaCl(aq), (CH3)4NCl(aq), and (C2H5)4NI(aq) at Different Ionic Strengths a. *J. Chem. Eng. Data* **2006**, *51*, 1660–1667, doi:10.1021/je060132t.
5. Li, N.; Wu, X.; Jia, W.; Zhang, M.C.; Tan, F.; Zhang, J. Effect of ionization and vehicle on skin absorption and penetration of azelaic acid. *Drug Dev. Ind. Pharm.* **2012**, *38*, 985–994, doi:10.3109/03639045.2011.635376.
6. Peira, E.; Carlotti, M.E.; Cavalli, R.; Trotta, M. Azelaic acid sodium salt in the formulation of microemulsions for topical applications. *J. Drug Deliv. Sci. Technol.* **2006**, *16*, 375–379, doi:10.1016/S1773-2247(06)50068-1.
7. Bertuzzi, A.; Gandolfi, A.; Salinari, S.; Mingrone, G.; Arcieri-Mastromattei, E.; Finotti, E.; Greco, A. V. Pharmacokinetic Analysis of Azelaic Acid Disodium Salt: A Proposed Substrate for Total Parenteral Nutrition. *Clin. Pharmacokinet.* **1991**, *20*, 411–419, doi:10.2165/00003088-199120050-00005.
8. Gupta, K.C.; Kumar, M.N.V.R. pH dependent hydrolysis and drug release behavior of chitosan/poly (ethylene glycol) polymer network microspheres. *J. Mater. Sci. Mater. Med.* **2001**, *2*, 753–759, doi:https://doi.org/10.1023/A:1017976014534.
9. Márquez, A.G.; Hidalgo, T.; Lana, H.; Cunha, D.; Blanco-Prieto, M.J.; Álvarez-Lorenzo, C.; Boissière, C.; Sánchez, C.; Serre, C.; Horcajada, P. Biocompatible polymer–metal–organic framework composite patches for cutaneous administration of cosmetic molecules. *J. Mater. Chem. B* **2016**, *4*, 7031–7040, doi:10.1039/C6TB01652A.
10. Simon-Yarza, T.; Baati, T.; Neffati, F.; Njim, L.; Couvreur, P.; Serre, C.; Gref, R.; Najjar, M.F.; Zakhama, A.; Horcajada, P. In vivo behavior of MIL-100 nanoparticles at early times after intravenous administration. *Int. J. Pharm.* **2016**, *511*, 1042–1047, doi:10.1016/j.ijpharm.2016.08.010.
